# The near-peer tutoring programme: embracing the ‘doctors-to-teach’ philosophy – a comparison of the effects of participation between the senior and junior near-peer tutors

**DOI:** 10.3402/meo.v20.27959

**Published:** 2015-09-08

**Authors:** Siaw-Cheok Liew, Chew-Fei Sow, Jagmohni Sidhu, Vishna Devi Nadarajah

**Affiliations:** 1 Department of Clinical Skills and Simulation Centre, International Medical University, Kuala Lumpur, Malaysia; 2 Teaching and Learning Office, Department of Human Biology, International Medical University, Kuala Lumpur, Malaysia

**Keywords:** clinical skills, medical students, undergraduate, Phase I, teaching

## Abstract

**Background:**

While there is an increasing pool of literature documenting the benefits of near-peer tutoring programme, little is known about the benefits for junior and senior peer tutors. Knowledge of the peer tutors’ perceived benefits at different levels of seniority will aid in the development of a near-peer tutoring programme that will better fulfil both curricula and personal aspirations of near-peer tutors. We, therefore, investigated the perceived benefits of participation in a near-peer tutoring programme for junior as well as senior near-peer tutors.

**Methods:**

Pre- and post-participation questionnaires were distributed to near-peer tutors after their clinical skills teaching sessions with Phase I undergraduate medical students. The Peer Tutor Assessment Instrument questionnaires were distributed to the 1) students, and to the 2) near-peer tutors (junior and senior) after each teaching and learning session for self-evaluation.

**Results:**

The senior near-peer tutors felt that their participation in the programme had enhanced their skills (*p*=0.03). As a whole, the near-peer tutors were more motivated (Pre 5.32±0.46; Post 5.47±0.50; *p*=0.210) to participate in future teaching sessions but did not expect that having teaching experiences would make teaching as their major career path in the future (Pre 4.63±1.07; Post 4.54±0.98; *p*=0.701). The senior near-peer tutors were evaluated significantly higher by the students (*p*=0.0001). Students’ evaluations of near-peer tutors on the domain of critical analysis was higher than self-evaluations (*p*=0.003).

**Conclusions:**

Generally, the near-peer tutors perceived that they have benefited most in their skills enhancement and these near-peer tutors were scored highly by the students. However, senior near-peer tutors do not perceive that the programme has a lasting impact on their choice of career path.

The emphasis of the General Medical Council (GMC) of the United Kingdom on the need for training medical students with teaching and learning skills encourages future doctors to become better teachers. The medical graduates must be able to ‘demonstrate appropriate teaching skills’, and as future physicians, they would need to understand the principles of medical education, be able to apply the various methods of teaching and learning, and be responsible to teach their colleagues (1). The irony of physicians requiring teaching skills and yet not been given formal or adequate training was addressed with the introduction of a near-peer tutoring programme which created an opportunity for students to teach and participate in student training while in medical school (2–4). The near-peer tutoring programme has been reported as one of the effective methods of curriculum delivery and medical students have found the programme beneficial to their learning (5, 6).

Many studies reported that near-peer tutoring is effective, cost efficient, and is a popular educational intervention tool where students themselves are involved in imparting educational principles and knowledge. However, the capability of students to teach each other and junior students successfully relied heavily on the training of these students as tutors (7–10). The evidence suggests that the reasons behind the success and the popularity of this programme were the academic benefits of near-peer tutoring programmes for the tutors and also the opportunity for students to have teaching experience (7, 8, 11–14).

Literature on the effect of near-peer tutoring on the near-peer tutors themselves suggested that the programme could increase the development of self-esteem, with near-peers becoming more effective communicators and having a better understanding of learning and teaching principles (15–19). While the literature on the benefits of near-peer tutoring is vast, none has compared the effect of near-peer tutoring between junior and senior near-peer tutors. Studies available, in isolation, looked at the effect of near-peer tutoring on the more senior near-peer tutors after the completion of the near-peer tutoring programme (15–22). It is important to determine the differences in skills, expectations, and motivation amongst the two groups of senior and junior tutors for the future development of a near-peer tutoring programme that will fulfil both curricula and personal aspirations of the near-peer tutors. Our university introduced a near-peer tutoring programme in 2012, and the primary aim of this study was to investigate the benefits of the near-peer tutoring programme for junior near-peer tutors (Year 3 students) as well as the senior near-peer tutors (recent graduates) in the domain of skills, expectation, and motivation. We also aim to compare 1) the medical students’ (Year 1 and Year 2) perceptions of their junior and senior near-peer tutors’ teaching skills and 2) the medical students’ (Year 1 and Year 2) evaluation of their junior and senior near-peer tutors’ clinical teaching with the near-peer tutors’ own self-evaluation scores.

## Methods

This study was conducted over a course of 8 months (September 2013 to May 2014) at the Clinical Skills and Simulation Centre, International Medical University (IMU), Kuala Lumpur, Malaysia.

### Sample size estimation

All the Year 1 and 2, Phase I, undergraduate medical students who were taught by the near-peer tutors were recruited for this study. The study population is made up of 35 junior and 16 senior near-peer tutors. For the junior near-peer tutors, a minimum of 19 students would need to be recruited as participants from the pool of near-peer tutors in the programme to achieve a 95% confidence level with an allowable error margin of ±7%. For the senior near-peer tutors, a minimum total of 15 students would need to be recruited as participants to achieve a 95% confidence level with an allowable error margin of ±0.1%.

### Inclusion and exclusion criteria

All the post-Year 3 medical students who have sat and passed their first Professional Examination were included in this study and categorised as the junior near-peer tutors. All medical students who have sat and passed their final year, newly graduated doctors waiting for their housemanship (equivalent to junior doctor or internship placements) at government hospitals, were included in this study and categorised as senior near-peer tutors. Participation was voluntary. All the junior and senior near-peers who gave informed consent were recruited for the study, and all those who did not give informed written consent were excluded.

All post-Semester 5 and post-Semester 10 medical students were invited to apply for the near-peer tutoring programme. The potential near-peer tutors were invited to attend a compulsory 1 day training workshop where they were taught on adult learning and clinical skills learning. After the workshop, the potential near-peer tutors were assessed on their clinical skills teaching. They were to teach two junior medical students on a selected clinical skills, and their teaching skills were evaluated by the clinical skills lecturers. The acceptance rate of these medical students for the near-peer tutoring programme after clinical skills lecturers’ evaluation was about 80%.

### Sampling method

The near-peer tutors (junior and senior) were given pre-participation questionnaires to fill prior to their teaching experiences at the clinical skills and simulation centre. The near-peer tutors were given four clinical skills sessions each, and each teaching session lasted 2 hours. The skills taught were either physical examination skills or procedural skills. At the end of the fourth session, the near-peer tutors were given post-participation questionnaires to fill.

The pre- and post-participation questionnaires (Peer Tutors Own Assessment) used in the assessment of the near-peer tutors were the adapted version of questionnaires developed by Buckley and Zamora (15). The questionnaire had 14 items with responses in the Likert-type scale of 1 (totally disagree) to 6 (totally agree). The pre-participation questionnaire (Appendix 1) explored the near-peer tutors’ perceived benefits and motivations to participate in the near-peer tutoring programme in three separate domains, namely 1) skills, 2) motivation, 3) and expectation prior to the teaching sessions. Likewise, the post-participation questionnaire (Appendix 1) explored the perceived benefits of their participation in the near-peer tutor programme after the teaching sessions. The Cronbach's alpha was 0.801 for the domain on skills enhancement, 0.714 for Motivation, and 0.814 for Expectations.

All the Year 1 and 2, Phase I, undergraduate medical students who were taught by the near-peer tutors were given the Peer Tutor Assessment Instrument questionnaire to fill. The questionnaires were distributed to the medical students at the Clinical Skills and Simulation Centre at the end of every class taught by the near-peer tutors. The adapted version of the Peer Tutor Assessment Instrument (Appendix 2) developed by Papinczak was used to assess the acceptability of the peer tutor teachings and was distributed to the Year 1 and 2 medical students to assess the skills taught by the near-peer tutors (16). The same questionnaire was also distributed to the near-peer tutors to assess their own performance in teaching their juniors.

The purpose of the study was explained to the students and consents were sought before the distribution of the hard copies of the questionnaires. The students were given 15 min each to complete the questionnaire.

### Ethical consideration

The study protocol was approved by the Joint Ethics Committee of the International Medical University in compliance with the Helsinki Declaration (Project Number: IMU 281/2013).

### Statistical analysis

Statistical Package for Social Sciences (SPSS) version 17.0 was used to analyse the data collected. The completed pre- and post-teaching questionnaires (Peer Tutors Own Assessment) by the near-peer tutors were evaluated using the independent as well as the paired *t*-test. The questionnaire completed by the students which assessed their acceptability of the teaching by the near-peer tutors (Peer Tutor Assessment Instrument) were evaluated using the independent *t*-test. In this study, a *P*-value of less than 0.05 was considered as statistically significant.

## Results

### Baseline data

Table 1 shows the demographic data of the near-peer tutors that participated in this study (*n*=34). Nineteen junior and 15 senior near-peer tutors were recruited for the study. The overall mean age ±SD of the participants was 21±2.75 years. An equal number of male and female participants were recruited (50%). Majority of the near-peer tutors did not have any previous teaching experiences (76.5%).

**Table 1:** Demographic data of the near-peer tutors

	Junior (*n*=19)	Senior (*n*=15)	Overall (*n*=34)
No. of volunteers (%)	19 (55.9)	15 (44.1)	34 (100.0)
Age (mean±SD)	21±2.14	24±20.5	21±2.75
Gender			
Male (%)	10 (52.6)	7 (46.7)	17 (50.0)
Female (%)	9 (47.4)	8 (53.3)	17 (50.0)
Previous teaching experience			
Yes (%)	6 (31.6)	2 (13.3)	8 (23.5)
No (%)	13 (68.4)	13 (86.7)	26 (76.5)

### Comparison of the pre- and post-participation scores

Table 2 shows the comparison of junior and senior near-peer tutors’ pre- and post-participation scores in the near-peer tutoring programme. The junior near-peer tutors (*n*=19), as analysed from the post-participation scores, indicated that the near-peer tutor programme had the most impact on their motivation to do more teaching sessions in the future and to help their junior peers (5.53±0.48). The senior near-peer tutors (*n*=15) initially reported lesser expectation on the skills enhancement (5.07±0.80) and have shown less motivation (5.27±0.58) compared with their junior counterparts prior to joining the near-peer tutoring programme. However, in the post-participation questionnaires, the senior near-peer tutors were more inclined to report that the participation in this programme had enhanced their skills the most (*p*=0.03). Prior to participation, the senior near-peer tutors expected that participation in this programme were more likely to help to enhance their curriculum vitae (CV) and indicated that teaching would be a major component in their career path (4.93±1.02) when compared with the responses from the junior near-peer tutors (4.39±1.07). Post-participation, the senior near-peer tutors reported a lesser degree of agreement than previously stated: that the programme had helped to boost their career prospects (4.67±0.75).

**Table 2:** Comparison of teaching scores

	Pre-teaching	Post-teaching	*T*-test (*p*-value)
(1) Junior peer tutors			
Skills (Mean±SD)	5.41±0.51	5.46±0.50	0.695
Motivation (Mean±SD)	5.37±0.36	5.53±0.48	0.169
Expectation (Mean±SD)	4.39±1.07	4.43±1.13	0.813
(2) Senior peer tutors			
Skills (Mean±SD)	5.07±0.80	5.47±0.53	0.030
Motivation (Mean±SD)	5.27±0.58	5.40±0.52	0.272
Expectation (Mean±SD)	4.93±1.02	4.67±0.75	0.292
(3) Overall (Junior and senior peer tutors)			
Skills (Mean±SD)	5.26±0.67	5.47±0.51	0.156
Motivation (Mean±SD)	5.32±0.46	5.47±0.50	0.210
Expectation (Mean±SD)	4.63±1.07	4.54±0.98	0.701

* *p*<0.05 was considered as statistically significant.

The male near-peer tutors in the pre-participation questionnaire responded that they were more likely to expect this programme to benefit them through skills enhancement (5.31±5.79) and to boost their career prospects (4.90±0.90) compared with the female near-peer tutors.

### Students’ evaluations and peer tutors’ self-evaluations

Table 3 shows the 1) students’ evaluation of their near-peer tutors post-teaching and learning sessions and 2) the near-peer tutors’ self-evaluations of their own clinical skills teaching. A large number (*n*=1,053) of Phase I medical students (Year 1 to Year 3) participated and gave their evaluations on their near-peer tutors’ teaching sessions (*n*=34) using the Peer Tutor Assessment Instrument. Of all the near-peer tutors recruited for the study (junior *n*=19, senior *n*=15), only 13 senior near-peer tutors responded and completed the Peer Tutor Assessment Instrument after they had completed their teaching sessions. Therefore, the students’ evaluation of the two senior near-peer tutors who did not complete the self-evaluations were removed. The Phase I medical students’ (Year 1 to Year 3) respondents’ data (n=985) was analysed for this study.

**Table 3:** Comparison of the students’ and self-evaluations

	Junior peers (*n*=19)	Senior peers (*n*=13)	*t*-test (*p*-value)	Senior and junior peers (*n*=32)	Paired sample *t*-test (*p*-value)
(1) Responsibility and respect					
Students	5.25±0.61	5.45±0.67	0.0001	5.31±0.31	0.694
Self	5.32±0.57	5.19±0.51	0.534	5.27±0.54	
(2) Information processing					
Students	5.09±0.78	5.38±0.77	0.0001	5.17±0.44	0.725
Self	5.09±0.60	5.18±0.48	0.649	5.13±0.55	
(3) Communication					
Students	5.14±0.81	5.41±0.82	0.0001	5.23±0.41	0.232
Self	4.89±0.79	5.27±0.53	0.146	5.05±0.71	
(4) Critical analysis					
Students	5.02±0.86	5.24±0.89	0.0001	5.07±0.38	0.003
Self	4.44±0.86	4.67±0.77	0.449	4.53±0.82	
(5) Self-awareness					
Students	5.02±0.86	5.24±0.89	0.0001	5.07±0.38	0.718
Self	5.16±0.67	5.08±0.73	0.748	5.13±0.68	

* *p*<0.05 was considered as statistically significant.

The senior near-peer tutors were evaluated significantly higher in all the domains assessed (Responsibility and Respect, Information Processing, Communication, Critical Analysis and Self-Awareness) when compared with their junior counterparts. There were no significant differences in the comparison of the self-evaluation scores of the senior and the junior near-peer tutors in all the domains assessed. Both groups scored lowest for the domain on critical analysis although the senior near-peers scored themselves slightly higher than the junior near-peers. Significant difference (*p*=0.003) was seen in the scores given by the students compared with the near-peer tutors’ (junior and senior) self-evaluations on critical analysis.

## Discussion

This study compares the effects of participation in the peer tutoring programme between the junior and the senior near-peer tutors. In particular, we looked at the effects of participation in several domains including skills, motivations, and their expectations. There is an increasing body of evidence suggesting that near-peer tutoring programmes are beneficial to the tutors as well as to the students (17, 20, 23). The results of our study aligned with the findings of Buckley and Zamora that all near-peer tutors, regardless of their seniority, perceived greater benefits from this programme towards the enhancement of their skills, which in turn motivated them to involve more in teaching in the future (15). The finding of this study shows increased motivation for near-peer tutors to involve in future teaching sessions. This finding suggests that participation in near-peer tutoring is associated with a desirable, positive inclination and attitudes towards teaching. There were also other reports that suggest similar gains of positive desirable attitudes amongst the senior students or junior doctors through experiences in teaching their junior colleagues in programmes similar to ours (24, 25).

In this study, the greatest benefit of participation in the programme perceived by the senior near-peer tutors was enhancement of their skills. This might suggest that the senior near-peers would have seen this programme as an opportunity to reinforce their basic clinical skills prior to their housemanship (equivalent to the junior doctor or internship placements in hospitals). Previous reports have identified that participation in a teaching programme such as this was perceived by near-peer tutors to be beneficial in the revision of their knowledge and provided them with an opportunity to reflect on their shortcomings, especially with regards to their knowledge depth. Additionally, participation in the programme helped to substantially provide them with a deeper level of understanding concerning the content of the knowledge taught (2, 26). Another study reported that near-peer tutors reported their increased confidence in clinical examination skills after teaching similar skills to others (17).

Although in general the near-peer tutors did see the importance of teaching and were more motivated to take up future teaching sessions, the senior near-peers did not feel that the programme had any effect on their choice of career paths or have any impact on the enhancement of their CV. It is therefore important to create more awareness of this career option by encouraging medical students to interact with more clinical teachers, introduce more formal teaching posts, and to expose them to this career path (27–29).

The near-peer tutors’ scores on the pre- and post-participation questionnaires were on the higher end of the Likert scale with an average mean of five out of six points. The voluntary nature of the recruitment of near-peer tutors for this programme would have drawn in near-peer tutors that were highly motivated to teach when they first participated. Similarly, Wadoodi and Crosby did highlight in their article that voluntary recruitment processes rather than selecting those who are academically sound would draw and create opportunities for participation from potential near-peer tutors who could be academically weak but truly motivated to improve their clinical skills (30). There were many debates on how the recruitment process should be done; however, the essence of the programme was about the mutual benefit for both tutors and students (6).

Although the students scored their near-peer tutors very highly on the critical thinking skills, the near-peer tutors did not score themselves as high. This finding suggests that either some of the near-peer tutors have set possibly higher standards for themselves or some did not perform the teaching steps related to the domain of critical thinking. Although not reported in relation to near-peer teaching, there are contradicting reports from the literature on students’ ability to critically think. These reports suggest that in higher education, critical thinking skills either improve as the students increase in seniority (31–34) or there is no improvement at all (35–37).

In the overall results, the students evaluated their near-peer tutors highly, indicating that students accept and recognise the teaching skills of the near-peer tutors. The large number of students’ response (*n*=985) in the evaluation of their near-peer tutors and the consistency of high scores across the various near-peer tutoring sessions also suggested that students benefited from learning clinical skills from their near-peer tutors. Likewise, the high self-evaluation scores of their own teaching reflected that the near-peer tutors also have much confidence in the delivery of their teaching sessions. In fact, in some studies, it had been shown that near-peer tutors benefited more academically and cognitively when compared with their students (12, 14, 38). There were other studies which also have shown evidences that students and tutors share many positive appraisals of near-peer tutoring (39–41). This result, therefore, reflects the GMC's aim of enriching the teaching portfolio in medical students (1, 42, 43), in accordance with the most updated GMC Good Medical Practice guideline (44).

The limitations of this study are the relatively small sample sizes of near-peer tutors and the subjectivity of scorings of the questionnaires given due to the probable differences in the interpretation of the test items by the near-peer tutors as well as the students. Therefore, the results should be interpreted within these limitations. However, the results do imply that the near-peer tutoring programmes benefited the near-peer tutors equally regardless of their seniority but had not changed their perceptions of incorporating teaching as a long-term career consideration in the future. Efforts should be taken to optimise the exposure to teaching in medical schools to create awareness and interest to this career path. Although the near-peer tutors reported that the near-peer tutoring programme have enhanced their skills and the students’ responses stated likewise, there is a need to prove the effectiveness of this programme where their actual performance may need to be formally evaluated by faculty members and further analysed.

## Conclusions

Both groups of near-peer tutors equally benefited from the near-peer tutoring programme regardless of their seniority. Near-peer tutoring enhanced the skills domain of the near-peer tutor but formal analyses of their performances are needed to prove its effectiveness. The teaching skills of the near-peer tutors were evaluated highly by the students; however, the senior near-peer tutors were rated significantly higher. Although near-peer tutors did see the importance of teaching, the programme did not have any influence on their choice of future career paths for the senior near-peer tutors. Future studies on junior doctors’ perceptions of teaching would be useful to determine if there is a need to nurture the passion for teaching at an early stage in medical school.

## Figures and Tables

**Table:** 

	Pre-teaching: *My purpose of becoming a peer tutor*	Post-teaching: *The peer tutoring sessions have*
Skills enhancement	1. Improve own learning skills	1. Improved own learning skills
	2. Improve practical teaching skills	2. Improved practical teaching skills
	3. Improve understanding of educational principles	3. Improved understanding of educational principles
	4. Increase confidence in speaking to groups	4. Increased confidence in speaking to groups
	5. Improve organisation/planning skills	5. Improved organisation/planning skills
Context (motivations)	1. Desire to help fellow students	1. Increased my desire to help fellow students
	2. Focus on practical skills	2. Helped me to focus on practical skills
	3. A good way to revise own clinical skills	3. Revised my own clinical skills
	4. Desire to emulate good teaching I have had	4. Increased my desire to emulate good teaching I have had
	5. Previous teaching experience encouraging me to do more	5. Encouraged me to do more
Career direction (expectations)	1. Teaching is an important part of my future career	1. I will undertake further teaching training
	2. Experience in teaching will help me to decide my career direction	2. Helped me to decide my career direction
	3. Teaching will be important – whatever my career route	3. Teaching will be a major part of my career
	4. Taking part will enhance my curriculum vitae	4. Enhanced my curriculum vitae

Adapted from Ref. (15).

**Table:** 

**Responsibility and respect**						
1. Completed all assigned tasks to the appropriate level	1	2	3	4	5	6
2. Completed all assigned tasks on time	1	2	3	4	5	6
3. Participated actively in the session	1	2	3	4	5	6
4. Showed behaviour and input that facilitated learning	1	2	3	4	5	6
5. Was punctual to the session	1	2	3	4	5	6
6. Listened and showed respect for the opinions of others	1	2	3	4	5	6
**Information processing**						
7. Brought in new information to share with the group	1	2	3	4	5	6
8. Provided information that was relevant and helpful	1	2	3	4	5	6
9. Seemed to use a variety of resources to obtain the information	1	2	3	4	5	6
**Communication**						
10. Was able to communicate ideas clearly	1	2	3	4	5	6
11. Made comments and responses that was not confusing	1	2	3	4	5	6
**Critical analysis**						
12. Gave input that was focussed and relevant to the case	1	2	3	4	5	6
13. Gave a summary of the session	1	2	3	4	5	6
14. Gave a summary of the session that showed evidence of reflection and evaluation	1	2	3	4	5	6
**Self-awareness**						
15. Appeared to the able to acknowledge his/her own strengths and weaknesses	1	2	3	4	5	6
16. Accepted and responded to criticism gracefully	1	2	3	4	5	6
**Other comments:**						





Adapted from Ref. 16.

## Appendix 1

Peer tutor's own assessment

Please answer the items below indicating strength of your agreement or disagreement with the statements by circling the number on the scale (1=totally disagree; 6=totally agree).

## Appendix 2

Peer tutor assessment instrument

Please answer the items below indicating the strength of your agreement or disagreement with the statements about the peer tutor's performance in the CSU session by circling the number on the scale (1=totally disagree; 6=totally agree)
